# Side-by-Side Comparison of Post-Entry Quarantine and High Throughput Sequencing Methods for Virus and Viroid Diagnosis

**DOI:** 10.3390/biology11020263

**Published:** 2022-02-08

**Authors:** Marie-Emilie A. Gauthier, Ruvini V. Lelwala, Candace E. Elliott, Craig Windell, Sonia Fiorito, Adrian Dinsdale, Mark Whattam, Julie Pattemore, Roberto A. Barrero

**Affiliations:** 1 eResearch, Research Infrastructure, Academic Division, Queensland University of Technology, Brisbane, QLD 4001, Australia; marieemilie.gauthier@qut.edu.au (M.-E.A.G.); lelwala.lelwala@qut.edu.au (R.V.L.); c.windell@qut.edu.au (C.W.); 2 Science and Surveillance Group, Post Entry Quarantine, Department of Agriculture, Water and the Environment, Mickleham, VIC 3064, Australia; candace.elliott@awe.gov.au (C.E.E.); julie.pattemore@awe.gov.au (J.P.); 3 Plant Innovation Centre, Post Entry Quarantine, Department of Agriculture, Water and the Environment, Mickleham, VIC 3064, Australia; sonia.fiorito@awe.gov.au (S.F.); adrian.dinsdale@awe.gov.au (A.D.); mark.whattam@awe.gov.au (M.W.)

**Keywords:** high throughput sequencing, plant virus and viroid detection, phytosanitary diagnostic assay, plant siRNA, post-entry quarantine facility

## Abstract

**Simple Summary:**

Increase in global trade represents new opportunities and challenges to the biosecurity system. The growth of imported plant materials into Australia, and associated pathogen testing, places pressure on limited resources to ensure the detection of pests of biosecurity concern at the border in a timely manner. Adoption of new technologies such as high throughput sequencing coupled with appropriate data analytical methods have the potential to accelerate quarantine testing. We report the use of a technology that relies on the plant host immune response against viruses and viroids to implement a single assay to scrutinize their presence across diverse plant commodities.

**Abstract:**

Rapid and safe access to new plant genetic stocks is crucial for primary plant industries to remain profitable, sustainable, and internationally competitive. Imported plant species may spend several years in Post Entry Quarantine (PEQ) facilities, undergoing pathogen testing which can impact the ability of plant industries to quickly adapt to new global market opportunities by accessing new varieties. Advances in high throughput sequencing (HTS) technologies provide new opportunities for a broad range of fields, including phytosanitary diagnostics. In this study, we compare the performance of two HTS methods (RNA-Seq and sRNA-Seq) with that of existing PEQ molecular assays in detecting and identifying viruses and viroids from various plant commodities. To analyze the data, we tested several bioinformatics tools which rely on different approaches, including direct-read, de novo, and reference-guided assembly. We implemented VirusReport, a new portable, scalable, and reproducible nextflow pipeline that analyses sRNA datasets to detect and identify viruses and viroids. We raise awareness of the need to evaluate cross-sample contamination when analyzing HTS data routinely and of using methods to mitigate index cross-talk. Overall, our results suggest that sRNA analyzed using VirReport provides opportunities to improve quarantine testing at PEQ by detecting all regulated exotic viruses from imported plants in a single assay.

## 1. Introduction

Pathogenic viruses and viroids infecting plants can result in significant economic and ecological losses. Preventing the introduction of such pests is therefore a fundamental concern to national biosecurity and border control agencies [1,2,3]. Traditional diagnostic tools such as serological or molecular assays (i.e., PCR, ELISA) require a priori knowledge of pathogens’ genetic blueprint to detect plant viruses and viroids. Other non-specific tests such as electron microscopy, woody indexing, or visual inspection do not require this prior knowledge [4,5,6]. However, they can result in false negatives/positives and generally provide a lower level of detection resolution that can be limited to pest features or higher-level taxonomy information (e.g., genus). Most of these traditional tests are also unable to detect multiple pathogens in a single assay [4,5,6]. Additionally, quarantined plants often require extended periods of time in post-entry quarantine (PEQ) (up to 2 years) which can significantly increase cost and risk to importers. Finally, a wide range of plant genera need to be tested at PEQ facilities, bringing an added complexity to their diagnostics testing requirements [6].

The use of high-throughput sequencing (HTS) technologies for detecting plant viruses started in 2009 [7,8,9]. Since then, these methods have become an integral part of research and diagnostics on plant viruses and viroids. They provide unbiased high-resolution detection of highly diverse viruses without needing prior characterization [5,10,11,12]. HTS also enables the identification of viruses that can cause either non-specific or no symptoms and are thus problematic for traditional diagnostics tools [13]. With increased affordability and portability of HTS, international groups and regulatory agencies are investigating its viability to rapidly screen for viral pathogens in plants in quarantine facilities, aiming to improve the rate at which propagation material is made available to producers. However, there is a need for harmonization and standardization of HTS-based virus diagnostics to accelerate quarantine testing. The number of different approaches in nucleic acid preparation, sequencing, and sequence data analysis highlights the challenges that the development of standard operating procedures may present, with several studies reporting discrepancies in results between different HTS or analytical methods [14,15,16]. Furthermore, challenges specific to HTS (e.g., adapter dimer formation, barcode misassignment, chimeric contig assembly) need to be carefully assessed [17].

While some studies have evaluated the sensitivity of specific HTS approaches compared to traditional tests in a given commodity [18,19], HTS technologies still need to pass rigorous tests and repeatedly demonstrate they are equal to, or better than the current sensitivity, specificity and reproducibility standards before being approved for routine use [11,20]. For this reason, HTS is not yet currently considered to be stand-alone replacements for traditional or molecular methods. Academic and governmental research groups both acknowledge the need for extant protocols to independently confirm the biological significance of detected viruses via HTS. Several international efforts are underway to critically catalogue methods available [17,21,22,23] and provide researchers and diagnostics laboratories with open-source reference datasets to compare and validate their detection pipelines [16,24]. There is also an increase in comparative studies reporting the performance of different approaches [14,15,16,21,25,26]. Together, this will help improve and accelerate harmonization, comparability, and reproducibility of HTS methods.

HTS methods use different nucleic acid fractions for detecting plant pests, with total RNA and small RNA (sRNA) being the two most widely used approaches for detection of viruses in plant tissues [17]. Compared to the total RNA approach, the sRNA method specifically targets small-interfering RNA (siRNA) and other functional sRNAs for sequencing, which has the advantage of biasing the detection method toward viruses and viroids that triggered a host immune response against these pathogens. Plant viruses produce double stranded intermediates during replication. The plant antiviral response involves distinct Dicer proteins that cleave these double-stranded forms of viral RNA into sRNAs that are 21 nucleotides (nt), 22 nt, and 24 nt long [23]. HTS techniques such as RNA sequencing (RNA-Seq) and sRNA sequencing (sRNA-Seq) yield short sequences of viral RNA or siRNA extracted from plants. These can be detected and identified using bioinformatics tools that can either perform a direct search on the sequenced reads or be assembled to generate longer sequences before annotation and diagnosis.

This study compares diagnostic results of molecular assays used at PEQ against both sRNA-seq and ribosomal depleted RNA-Seq methods. We use VirReport and other bioinformatics tools and pipelines to detect regulated viruses and viroids in imported plants under quarantine to assess the feasibility of adopting HTS testing as a routine diagnostics tool in PEQ facilities.

## 2. Materials and Methods

### 2.1. Plant Material Preparation and Nucleic Acid Extraction

Assays used at PEQ identified 14 imported plants infected with one or more endemic or regulated (i.e., deemed of biosecurity concern) viruses/viroids for comparison against HTS protocols. These plants comprised a diverse range of commodities, namely citrus (*n* = 6), iris (*n* = 1), ornamental grass (*n* = 1), prunus (*n* = 1), raspberry (*n* = 1), strawberry (*n* = 3) and sweet potato (*n* = 1) (Table 1). Three leaves of different ages from different parts of the plant were selected for each plant and combined for downstream processing. Tissue samples were collected using a single-use sterile blade or by hand with single-use gloves. Approximately 100 mg of leaf/midrib was subsampled for nucleic acid extraction.

Total RNA extracts were prepared with an automated Maxwell^®^ Rapid Sample Concentrator (RSC; Promega, Madisson, WI, USA) instrument using Maxwell^®^ RSC SimplyRNA Tissue kit (AS1340, Promega). In brief, tissue samples were placed with a sterile 7 mm stainless steel bead in a sterile 2 mL round bottom tube and stored at −80 °C for 30 min. The tubes were then placed into a TissueLyser (Qiagen, Germantown, Maryland, Germany) for 2 min at 50 Hz. Homogenization solution (600 µL) was added to each tube followed by 300 µL of lysis buffer. The tubes were subsequently vortexed and spun for 5 min at 20,000× *g* before loading 500 µL supernatant into the Maxwell RSC cartridge. The quantity of the RNA extracted was measured by fluorometry using a Qubit RNA BR Assay kit (ThermoFisher Scientific, Waltham, MA, USA) on a Quantus fluorometer (Promega) according to manufacturer’s recommendations.

### 2.2. Routine Diagnostics Assays

Virus and viroid infection was confirmed by PCR or RT-qPCR. Assay details described in Supplementary Materials Table S2.

### 2.3. Library Construction and Sequencing

Aliquots of 2 µg of the same total RNA extract was shipped on dry ice to two separate HTS providers referred here as service provider 1 (SP1) and 2 (SP2). Fourteen and nine RNA samples were submitted to SP1 and SP2, respectively where the RNA quality was assessed and sequencing libraries were prepared (Supplementary Materials Table S1). Samples with RNA integrity scores ≥ 7.0 (Bioanalyser 2100, Agilent, Santa Clara, CA, USA) were retained for library preparation.

- *Sequencing provider 1 (SP1)*

For RNA-Seq library preparation, samples were subject to rRNA depletion using the Ribo-Zero Plant kit (Illumina, San Diego, CA, USA) as per the instruction manual. Then, the TruSeq Stranded Total RNA kit (Illumina) was used to generate single-indexed libraries. A proprietary sRNA library preparation protocol was used that included two PAGE electrophoresis steps: the first one to separate 18 to 30 bp long RNA from total RNA before library preparation and a second one after library preparation to separate final PCR products in the range of 100–120 bp. The sRNA libraries generated used single-indexed sequencing adapters and unique molecular identifiers (UMIs). All samples were sequenced using a proprietary rolling cycle sequencing approach and a custom sequencing platform, generating 2 × 150 bp paired-end RNA-Seq reads and 75 bp single-end reads for the sRNA-Seq samples (Supplementary Materials Figure S1).

- *Sequencing provider 2 (SP2)*

The TruSeq Stranded Total RNA kit with Ribo-Zero Plant (Illumina) was used to produce dual-indexed RNA-Seq library libraries. Paired-end 2 × 150 bp reads were sequenced using an Illumina NovaSeq 6000. For sRNA library preparation, samples were processed with the NEBNext Multiplex Small RNA Library kit (New England Biolabs, Ipswich, MA, USA) with a final size selection performed on a Pippin prep system using 3% cassettes with the P marker and 125–175 bp size selection parameters. Single-end 75 bp reads were sequenced on an Illumina NextSeq 500 (Supplementary Materials Figure S1).

### 2.4. Bioinformatics Analyses

All raw fastq files were deposited in the Short Read Archive (SRA) database under the BioProject PRJNA752836. Post-sequencing, reads generated by RNA-Seq were filtered for low quality regions and sequencing adapters using Trim Galore [27]. The adapters were the specific forward (5′-AAGTCGGAGGCCAAGCGGTCTTAGGAAGACAA-3′) and reverse (5′-AAGTCGGATCGTAGCCATGTCGTTCTGTGAGCCAAGGAGTTG-3′) adapters for the SP1 samples; and Illumina TruSeq adapter (5′-AGATCGGAAGAGC-3′) for the SP2 samples. The sRNA dataset provided by SP1 was already filtered for low quality sequences and adapters using SOAPnuke [28]. We performed an additional filtering step for low quality regions using Fastp [29]. The sRNA reads provided by SP2 were clipped for Illumina adapters and low quality regions using Fastp. FastQC was used to check the quality of reads prior to and after quality filtering (≥Q30) for both RNA-Seq and sRNA-Seq samples [30]. The amount of rRNA was assessed by aligning the reads with Bowtie [31] against the Silva database (https://www.arb-silva.de, accessed on 9 January 2021).

Four bioinformatics approaches were executed on the ribodepleted RNA-Seq samples to detect viruses and viroids. We trialed two methods that use direct annotation on quality trimmed reads without any assembly: the plant virus detection pipeline (PVDP) [32] and Kodoja [33]. These workflows were run on default parameters using the pre-computed database kodojaDB_v1.0 and the PlantVirusesDB_0420v4_masked.fa for Kodoja and PVDP, respectively. We used the stringent level assignment for viruses (i.e., same assignment designated by Kraken [34] and Kaiju [35]) and the non-stringent assignment for viroids (as viroids do not have protein assignments in RefSeq). We also tested two de novo transcriptome assemblers: the rnaviral mode of SPAdes 3.15 [36] and Trinity-v2.11.0 [37] using singularity. For Trinity, the quality-trimmed reads were filtered using a removal list of diverse non-informative sequences including ribosomal RNAs and representative host plant chloroplasts and mitochondria, before performing de novo assembly following best practice recommendations for this tool (https://informatics.fas.harvard.edu/best-practices-for-de-novo-transcriptome-assembly-with-trinity.html, accessed on 9 January 2021). SPAdes 3.15 was run using the rnaviral mode on default parameters, using k-mer lengths of 33 and 49. The contigs obtained from the de novo assembly approaches were collapsed into scaffolds using CAP3 [38]. Nucleotide sequence similarity search of scaffolds was then performed against the NCBI nucleotide (nt) database using megablast [39]. The top 5 matches were retained, and a report table was obtained using BlastTools (https://github.com/schmidda/blast-tools, accessed on 9 March 2021) (Supplementary Materials Figure S1).

We compared the detection of viruses and viroids by the sRNA-Seq technology using two methods: VirusDetect 1.7 [40] and VirReport (https://github.com/eresearchqut/virreport.git, accessed on 2 February 2022), a portable, scalable and reproducible nextflow [41] pipeline which uses a similar framework to the Yabi web-based Virus Surveillance and Diagnostics pipeline [42] (Supplementary Materials Figure S2). Before running VirusDetect, reads were first filtered for ribosomal RNA and host representative sequences (mitochondria and chloroplast) as recommended by the developer. The software was run using the cut-off depth parameter of 5 (default). VirReport was tested using two different de novo assemblers: SPAdes 3.14 [43,44]. and Velvet 1.2.10 [45] (Supplementary Materials Figure S1). The downstream results obtained from these assemblers are referred to as VirReport-SPAdes and VirReport-Velvet from here onwards). For SPAdes, several k-mer ranges were tested and the optimal setting which maximized recovery of viruses and viroids for this study was 9 to 21, increasing by increments of 2 (data not shown). For Velvet, a k-mer size of 15 and a depth cut-off of 3 was used. For both VirusDetect and VirReport, we restricted the input for de novo assembly to 21–22 nt- or 24 nt-long reads. The contigs obtained by SPAdes and Velvet were blasted against NCBI as per protocol for the RNA-Seq data. A custom python script was applied to derive the top virus or viroid blastn hit (from the report table derived with BlastTools), and use it as a reference to map the filtered 21–22 nt- and 24 nt-long reads using Bowtie, allowing up to a single mismatch (which is equivalent to up to 5% sequence divergence among mapped reads onto the same reference genome). PCR duplicates were identified using UMICollapse [46] for the libraries which incorporated UMIs for the sRNA samples from the SP1. From the obtained alignments, we calculated the total number of aligned reads and deduplicated reads (for samples from SP1). We normalized counts using reads per million (RPM): total number of aligned reads × 1,000,000/total number of quality-filtered reads. Coverage and average depth were calculated using Picard tools (http://broadinstitute.github.io/picard/, accessed on 9 March 2021).

To evaluate the concordance of the de novo assembled viral contigs across methodologies and enable the identification of artefacts such as chimeric contigs, we selected, and where applicable derived, representative sequences for each detected viruses and viroids using the de novo contigs generated from both ribodepleted RNA-Seq and sRNA-Seq. For cases where a suitable representative sequence was not available, we used a public reference genome. To compare read abundance for a given virus or viroid across sequencing technologies, the representative sequences were used as references to map high quality reads from virus-derived reads from each sample for both HTS approaches, to derive read counts and fragments per kilobase of transcript per million mapped reads (FPKM).

To compare the performance of each method and HTS technology combination, we tested their ability to detect a total of 38 known target viruses and viroids species identified by PEQ across both sequencing platform including 24 across 14 plant samples for the SP1 and 14 across nine samples for the SP2 (Table 1). We derived the number of true positive (TP) and false positive (FP) detections and calculated sensitivity, which corresponds to the number of TPs (number of correctly identified viruses and viroids) divided by the total number of known target species [16,47]. To normalize reads from each sample and HTS approach, both sRNA-Seq and RNA-Seq derived quality trimmed reads, which were filtered from non-informative RNA products, were randomly subsampled using seqtk [48]; the sRNA-Seq dataset was also limited to reads ranging in size from 18 to 25 nt. The chosen subsample data sizes were 1,000,000 (1 M), 2.5 M and 4 M single reads for the sRNA-Seq data; and 1 M, 2.5 M, 4 M and 10 M paired-end reads for the RNA-Seq datasets. These subsamples were reanalyzed using Kodoja (RNA-Seq) and VirReport-Velvet (sRNA-Seq). For each subset of data, we derived the number of TPs and FPs, and calculated sensitivity and false discovery rate using the formula FP/(FP + TP) [16,47].

We also implemented a contamination flag (CF) which was applied on Kodoja, PVDP, VirReport and VirusDetect. Candidate contamination events were flagged based on RPM value reported for each target detected in the final virus table output. We used RPM values derived by the bioinformatics method (PVDP, VirusDetect) or derived it independently (Kodoja, VirReport). The CF assumptions are that: (1) a virus or viroid present in high titer in a given sample is likely to be the source of contamination in other multiplexed samples in the same run, and (2) detection of reads matching to this pathogen in other samples occur at a significantly lower abundance. We first calculate the maximum RPM value recorded for each virus and viroid identified on a run. If for a given virus, the RPM value reported for a sample represented less than a percentage of this maximum RPM value, it was then flagged as a contamination event. We tested 0.1, 1 and 10% as minimum threshold values.

## 3. Results

### 3.1. General Sequencing Statistics

In this study, we used a set of quarantined positive control PEQ reference plants including citrus, prunus, sweet potato, raspberry, strawberry, and an ornamental grass infected with known endemic and regulated (i.e., of biosecurity concern) viruses and/or viroids (Table 1). We tested these plants using two HTS approaches, ribodepleted RNA-Seq and sRNA-Seq. HTS data for both methods were generated independently by two service providers (SP1 and SP2) using distinct library prep and sequencing approaches.

**Figure 1:**
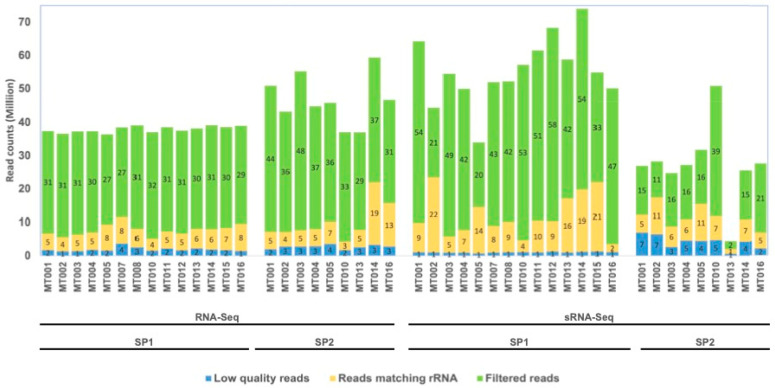
Distribution of RNA reads collected across samples. Bar chart showing amount of filtered reads in millions that were left-over (in green) after removing low-quality reads (in blue) and reads matching ribosomal RNA (in yellow) for RNA-Seq (on the left) and sRNA-Seq methods (on the right). The counts were split between sequencing providers (SP1 and SP2).

For ribodepleted RNA-Seq datasets, we obtained, on average, 37.9 and 46.7 million paired-end reads by SP1 and SP2, respectively. Quality filtering removed on average 5% and 6% of the reads produced by SP1 and SP2 samples, respectively. To assess the effectiveness of ribosomal depletion, quality trimmed reads were mapped onto the Silva database showing varying levels of retained rRNAs across samples, ranging from 13 to 29% for SP1 and 7 to 33% for SP2. On average, rRNAs accounted for 20% and 16% of the paired-end reads produced by SP1 and SP2, respectively (Supplementary Materials Table S2). There was no correlation between the plant species and the level of residual rRNA present across samples (Figure 1 and Supplementary Materials Table S3).

From sRNA-Seq datasets, we produced on average 55.4 and 27.5 million single-end reads by SP1 and SP2, respectively. Quality filtering resulted in the removal of 15% of reads on average for the SP2 samples and 2% for the SP1 samples, the latter value expected for samples that were already quality-filtered by the sequencing provider. The rRNA content was highly variable in the sRNA samples, accounting for 7% to 53% of the quality-filtered reads in the SP1, compared to 15% to 49% in SP2 samples. There was an association between the commodity and the percentage of rRNA detected in the samples, with the grass, sweet potato, and citrus samples being the least affected while the strawberry, peach and raspberry showed a higher level of rRNAs across both the SP1 and the SP2 samples (Figure 1 and Supplementary Materials Table S3). These rRNAs mainly originated from plants, but we also recovered matches to insects such as scale insects and aphids (data not shown).

### 3.2. Small RNA HTS Data Generates Fewer and Targeted De Novo Assembled Scaffolds

Detection of plant viruses and viroids by distinct HTS methods requires consideration of the intrinsic differences between these methods to detect pest sequences among host RNAs. RNA-Seq is a generalist approach that detects the presence of viral sequences along with protein-coding and long non-coding host RNA sequences. For the RNA-Seq samples, the SPAdes workflow recovered on average 81,681 contigs from the SP1 samples and 76,909 contigs from the SP2 samples (post CAP3 scaffolding). In comparison, the Trinity assemblies contained approximately 25% more contigs, with an average of 108,492 contigs from the SP1 samples and 102,901 contigs from the SP2 samples (Supplementary Materials Table S4A).

**Figure 2:**
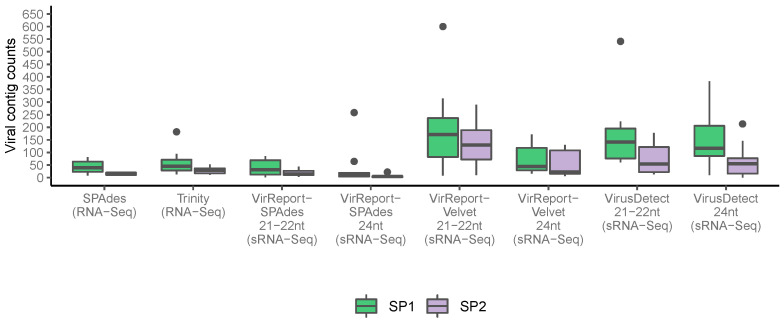
Total number of viral contigs recovered for each program tested that uses an assembly-based approach. The counts are split between sequencing providers (SP1 in green and SP2 in purple). The boxplot limits indicate the first and third quartiles, with the central line marking the median. Vertical lines extending from each box capture the remaining data that sits within 1.5 times of the interquartile range, while the dots placed past the line edges denote outliers.

In contrast to RNA-Seq, sRNA-Seq datasets generated a few dozen to several thousand scaffolds. The size of the assembly was highly dependent on the sequencing technology, the method, and assembler used. The assemblies contained significantly more contigs in the samples from SP1 than from SP2 (*p* < 0.001), and both Virus Detect and VirReport-Velvet generated more contigs than VirReport-SPAdes (Supplementary Materials Table S4A). After performing homology searches against the non-redundant NT database, we only retained contigs with sequence similarity to viruses and viroids. We recovered twice as many viral contigs in the SP1 samples compared to the SP2 samples across sequencing platforms and bioinformatics tools tested. For the RNA-Seq samples, the number of viral contigs recovered by RNA-Seq was on par between SPAdes and Trinity. However, in the sRNA-Seq samples, the assemblies derived using either VirusDetect or VirReport-Velvet contained more contigs than derived using VirReport-SPAdes (Figure 2 and Supplementary Materials Table S4B).

### 3.3. Evaluating Sensitivity of Detection of Plant Virus and Viroids

**Figure 3:**
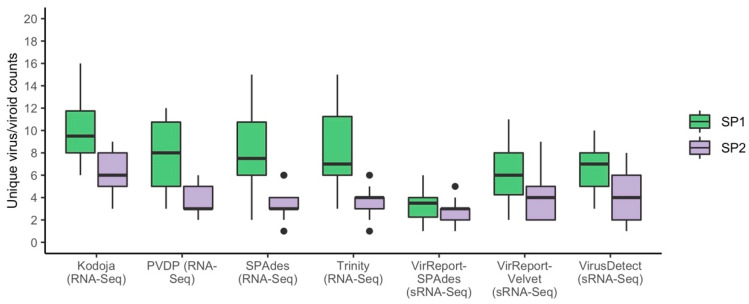
Side-by-side comparison of the total number of unique viruses and/or viroids detected by RNA-Seq and sRNA-Seq across samples by each software. The counts are split between sequencing providers (SP1 in green and SP2 in purple). See Figure 2 legend for boxplot interpretation.

Looking across both read-based and assembly-based software approaches, the total number of unique viruses and viroids recovered per sample varied significantly. The number of viral candidates detected in the SP1 samples was, on average higher than in the SP2 samples for both sequencing technologies (Figure 3). To measure the sensitivity of each method and HTS technology combination, we checked their ability to detect a total of 38 viruses and viroids across both sequencing platforms. Among the tested viruses, we identified a novel potyvirus in sample MT010 showing high sequence homology to both Sorghum mosaic virus and Maize dwarf mosaic virus, and tentatively named Miscanthus sinensis mosaic virus (MsiMV) (Le Blanc et al., submitted) (Table 1).

For the RNA-Seq samples, only Kodoja could detect all the viruses and viroids in the SP1 samples, while PVDP, SPAdes, and Trinity missed the detection of a Hop stunt viroid (HSVd) present in sample MT003. Kodoja only recovered two pairs of reads matching HSVd that harbored slight nucleotide differences to each other, explaining why the other methods failed to find it. PVDP, Kodoja, and SPAdes were able to detect all the viruses and viroids identified by PEQ in the SP2 samples, while Trinity failed to detect the HSVd present in the sample MT003. Thus, across the sequencing providers, Trinity achieved overall the lowest sensitivity (36/38, 94.7%), followed by SPAdes and PVDP (37/38, 97.3%), and finally Kodoja (38/38, 100%) (Table 2 and Supplementary Materials Table S5).

For the sRNA-Seq data, VirReport detected all the viruses and viroids from the data generated from both sequencing providers when using Velvet as the assembler (sensitivity of 100%, 38/38). It also recovered all targets for the SP1 when using SPAdes 3.14 but missed three targets in sample MT003 for the SP2 (sensitivity of 92.1%, 35/38). The MT003 sample had about half the number of reads of its SP1 counterpart, and with additional sequencing data gathered, VirReport-SPAdes was able to recover the missing viral targets (data not shown). VirusDetect detected all the viruses and viroids from the data generated from both sequencing providers and thus achieved an overall sensitivity of 100% (38/38) (Table 2 and Supplementary Materials Table S5).

For the sRNA data, the contigs generated by VirusDetect (which relies on Velvet for its de novo assembly step) and VirReport-Velvet were shorter than the ones generated by VirReport-SPAdes. However, the lower k-mer range (9–21) applied in VirReport-SPAdes meant that we observed many misassembled contigs in the derived assemblies. We could identify that these were caused by shared stretches of up to 20 nucleotides present in the host plant and virus sequences, yielding chimeric plant-virus contigs. SPAdes logs flagged a failure to determine erroneous k-mer threshold and estimate good valley values indicating that the lower bound of the k-mer range was likely yielding suboptimal results. The SPAdes software developers also stated that their tool had not been tested on sRNA reads and recommended not using k-mers below 15 to reduce the risk of misassembly.

Additional viruses detected through HTS include Citrus virus A in MT007 and MT008, Cherry rasp leaf virus in sample MT012, Strawberry vein banding virus in sample MT013, MT014, and MT015, Beet pseudoyellows virus in MT014, Sweet potato symptomless virus 1, and Sweet potato badnavirus in MT016. We also found rubus yellow net virus (RYNV) in samples MT005 and MT006 which are likely endogenous integrations into the host raspberry genome [49]. These were either not regulated pathogens of concern, or not regulated in the given host in which they were found.

### 3.4. Viral Abundance Highly Variable

We recovered a near-complete genome for most viruses and viroids targeted in this study using contigs obtained with Velvet, SPAdes, VirusDetect and/or Trinity, except for ISMV. The HSVd contig recovered by RNA-Seq for MT008 yielded a chimeric virus-host sequence misassembly that showed sequence similarity to two contigs assembled using sRNA-Seq data. Interestingly, only one of the sRNA-Seq derived contigs matched the complete HSVd genome, while the second showed sequence divergence explaining why they did not assemble into a single contig. Additionally, we identified four CTV strains infecting sample MT004 and two distinct TRSPV RNA2 in sample MT012. These derived consensus sequences were used to map back reads to estimate the virus-derived read abundance. Only for ISMV, we used its best sequence homology hit as mapping reference (KT692938.1). There was a high variation in the number of reads recovered for viroids or viruses ranging from a couple to several millions of reads. The MsiMV present in MT010 was the most abundant virus detected by all HTS technologies and service providers. Viral abundance otherwise differed between sequencing technologies, highlighting that the most abundant viral small interfering RNAs which accumulate as a result of plant immune response do not necessarily correspond to the viruses or viroids detected as the most abundant in the host plant, via isolated total RNA (Supplementary Materials Table S6).

### 3.5. Detection of Cross-Sample Contamination Events

**Figure 4:**
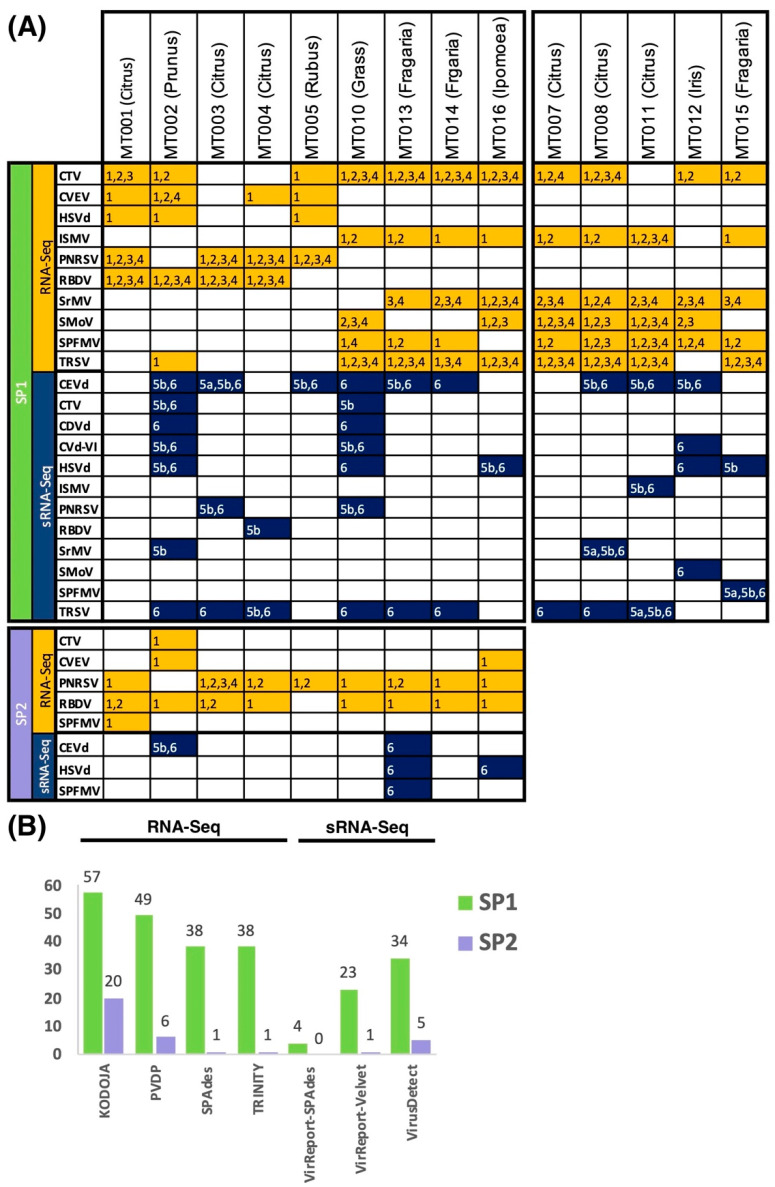
Detection of false positive viruses and viroids across samples. (**A**)**.** Details of all predicted viral contaminants detected in each sample sequenced by SP1 (top) and SP2 (bottom). Each number in the table corresponds to one of the software tested: numbers 1 to 4 refer to methods applied to RNA-Seq data; numbers 5 to 6 refer to methods applied to sRNA-Seq data. 1 = Kodoja, 2 = PVDP, 3 *=* SPAdes, 4 = Trinity, 5a = VirReport-SPAdes, 5b = VirReport-Velvet, 6 = VirusDetect. (**B**). Total number of false positive events detected per method and sequencing technology tested across 14 plant samples for the SP1 samples and 9 samples for the SP2 samples. The CVD-VI call detected by VirReport-Velvet in sample M010 was only detected in the de novo assembly derived using 24 nt-long reads.

Some of the viruses and viroids identified in control plants (Table 1) were detected in other multiplexed samples in the same RNA-Seq and sRNA-Seq experiments. These cases were either found in a commodity that is not known to be a host to the virus/viroid or in a known host, but PEQ molecular assays did not confirm its presence. Our samples were processed with Vitis samples on the same sequencing run but these were not part of our pilot study. Thus, any detection of pathogens specific to grapes across our samples were ignored. Figure 4 depicts identified cross-sample contamination events found for each HTS technology and service provider. For the RNA-Seq samples processed by SP1, each tool detected events across all 14 samples; Kodoja reported a total of 57, PVDP 49, SPAdes 38, and Trinity 38 events, respectively. For those processed by the SP2, Kodoja identified 20 false-positive events across all 9 samples, PVDP 6 events across 5 samples, Trinity 1 event, and SPAdes 1 event. For the sRNA-Seq samples processed by the SP1, VirusDetect, VirReport-Velvet, and VirReport-SPAdes identified 34, 23, and 4 false-positive events, respectively. In comparison to the SP2 samples, VirusDetect, VirReport-Velvet, and VirReport-SPAdes detected 5, 1, and 0 cross-sample contaminations, respectively (Figure 4).

Citrus tristeza virus, ISMV, MsiMV, SMoV, SPFVM, and TRSV accounted for most false positive detections in the RNA-Seq samples processed by SP1, while PNRSV and RBDV were the most pervasive in SP2. In contrast, CEVd was the most prevalent false positive detection among sRNA-Seq data generated by SP1 (Figure 4A). Interestingly, the plants infected with this viroid showed the highest load of viroid-derived sRNAs (Supplementary Materials Table S6).

A contamination Flag (CF) was implemented for data derived by Kodoja, PVDP, VirusDetect, and VirReport. We found that depending on the bioinformatics tool and the service provider used, the minimum threshold percentage to detect false positives varied to either 0.1% (PVDP), 1% (Kodoja, VirReport-SPAdes, VirReport-Velvet, and VirusDetect for SP2 data), or 10% (VirusDetect for SP1 data). Application of false-positive cut-off thresholds to Kodoja results for the RNA-Seq data resulted in two out of the 57 index hoping events missed for SP1 datasets (Supplementary Materials Table S7), while none were missed for the SP2 samples (Supplementary Materials Table S8). We also detected all cross-sample contamination events for data derived by PVDP for the SP1 samples (Supplementary Materials Table S9) but one out of the six false positives was missed for the SP2 samples (Supplementary Materials Table S10). For the sRNA-Seq data, the CF identified all contamination events detected by VirReport-SPAdes (Supplementary Materials Table S11), VirReport-Velvet (Supplementary Materials Tables S12 and S13), and VirusDetect (Supplementary Materials Tables S14 and S15).

### 3.6. Subsampling Reduces False Discovery Rate

We hypothesized that using subsets of rRNA-filtered HTS data will minimize or prevent the detection of cross-talk events owing to the observed near background level of cross-sample contamination. Indeed, we found that subsampling of informative HTS data reduced the false-positive discovery rate for both viruses and viroids using Kodoja and VirReport-Velvet for RNA-Seq and sRNA-Seq samples, respectively (Table 3). Subsampling informative reads down to 1 M detected all true positive viruses, except when using VirReport-Velvet for SP1 generated data requiring at least 2.5 M sRNA-Seq reads. For viroids, 1 M or 2.5 M sRNA-Seq subsets detected all viroids using SP1 or SP2 datasets, respectively. In contrast, when using ribodepleted RNA-Seq data, 2.5 M or 10 M paired-end reads were needed to detect all known viroids using SP2 or SP1 datasets, respectively (Table 3).

## 4. Discussion

This study contributes to the growing body of research aiming to accelerate the validation and adoption of HTS-based diagnostic tools to detect and identify viruses and viroids in plant quarantine facilities. We selected and tested several plant commodities with varying disease profiles for regulated viruses and viroids established through molecular methods used at PEQ. We assessed the performance of two HTS methods (RNA-Seq and sRNA-Seq) prepared by two separate sequencing providers, as well as different bioinformatics analyses tools, in independently detecting and identifying these pathogens. Several studies have previously reported detection discrepancies between different HTS or analytical methods [14,15,16]. This study highlighted the need to carefully inspect the plant virus and viroid sequences detected using a given HTS technology, library prep chemistry, sequencing chemistry and bioinformatics analytical method. We found that for the RNA-Seq samples, recovery of all true positives depended on the sequencing chemistry and bioinformatics tool combination used, with some tools struggling to detect HSVd, present at low titer in sample MT003, from one sequencing provider. The direct annotation method Kodoja was the only tool among the four tested approaches to detect HSVd in RNA-Seq data generated by the two service providers. In comparison, the use of sRNA-Seq data detected all true positive viruses and viroids irrespective of the service provider and analytic tool used (i.e., VirusDetect and VirReport-Velvet). The relative enrichment of the viral signature in sRNA-Seq data, compared to ribodepleted RNA-Seq datasets that capture the expression of diverse host plant RNAs, is likely responsible for the ability of sRNA-Seq datasets to detect viroids with relatively low expression titer.

We detected differing levels of false positives in our dataset. Cross-contamination can occur at different steps of the laboratory process including nucleic acid extraction, library preparation, and sequencing. We hypothesize that the contamination observed in our study is due to index misassignment (cross-talk), which can be introduced by a variety of mechanisms, including: (a) oligo manufacture error or contamination; (b) index hopping, which is a phenomenon that can occur due to low levels of free index primers present in the sample multiplexed pool during capture enrichment; (c) “spreading of signal” on patterned flow cells that impacts Illumina instruments such as the NovaSeq, which uses with exclusion amplification chemistry; (d) sequencing errors [50,51,52,53]. Cross-sample contamination of RNA-Seq reads has been estimated to occur at frequencies ranging from 0.6 to 10% on Illumina instruments using patterned flow cells, resulting in appreciable false-positive detections for sensitive applications [54,55]. In comparison, both NextSeq 500 using non-patterned flow cells with random cluster chemistry and DNA nanoball (DNB)-based HTS achieve low levels of index misassignment [51,54,55].

We detected significant cross-talk events among RNA-Seq samples processed by SP1 (which used single indexes), contrasting with the lower cross-sample contamination observed for SP2 samples. Since DNB-based HTS has been reported to achieve exceptionally low index cross-talk with single indexing [51], we cannot ascertain what was the precise source of index misassignment in SP1 samples (e.g., use of single index, oligo synthesis errors/contaminations or sequencing error). The incorporation of unique molecular identifiers (UMIs) alone by SP1 for sRNA data minimized the number of duplicates in samples harboring highly abundant viruses and viroids but did not prevent cross-sample contaminations.

The addition of dual-indexed (combinatorial) adapter oligos flanking the RNA insert by SP2 mitigated but did not eliminate cross-contamination in RNA-Seq samples sequenced on a NovaSeq. We found that a combination of high titer pathogens and extensive sequencing depth were key contributing factors increasing false positive detections, as has been reported elsewhere [56]. The use of unique dual indexed sequencing adapters (UDIs) and/or UMIs is recommended to minimize index misassignment on such sequencing instruments [52]. Even though the library kit used by the SP2 for the sRNA samples did not incorporate UDIs or UMIs, these samples were sequenced on a non-patterned Illumina instrument (NextSeq 500), and as a result they harbored a comparatively low level of false-positive detections.

Looking across bioinformatics tools tested, methods based on direct read annotation and reference guided mapping identified more false positives across samples compared to de novo assembly approaches. Processing sRNA-Seq data generated by the SP2, using VirusDetect, VirReport-Velvet, and VirReport-SPAdes approaches detected 5, 1, or 0 false positives across samples, respectively. VirusDetect uses reference-guided mapping and de novo assembly approaches to identify viruses, and its increased detection of false positive events is mainly due to its reference mapping approach. The VirReport pipeline relies only on de novo assembly to detect viruses/viroids making it is less prone to false positive reporting. We used two assemblers (SPAdes and Velvet) with this pipeline and found that although SPAdes yields a lower amount of false positives than Velvet, it can result in chimeric host-virus assemblies and the failure to detect low titer viruses. In contrast, VirReport combined with Velvet, and VirusDetect detected all true positives. Therefore, we found that we achieved the highest overall performance for the set of samples tested in this study by using sRNA sequencing technology from the SP2 sequenced on NextSeq and analyzed using either VirReport-Velvet or VirusDetect. These combinations detected all expected true positives while minimizing the detection of false positives.

The viruses that contributed the most to false-positive events in the RNA-Seq datasets differed between SP1 and SP2. This observation is partly due to differences in samples in the SP1 pool, namely MT011 and MT012, not included in the SP2 set. Additionally, using dual indexes by SP2 and differences in hamming distances between indexes can influence how samples can be prone to cross contamination. CEVd was the most prevalent false positive in the sRNA-Seq dataset processed by SP1 and was likely caused by the presence of high titer viroid-derived sRNAs in two samples present in our dataset.

Subsampling of informative reads depleted from host ribosomal, chloroplast, and mitochondrial RNAs for RNA-Seq and sRNA-Seq datasets significantly reduced false discovery rates. However, we started losing sensitivity before we could eliminate false positives. PVDP reports useful parameters such as evenness, read dispersion, and certainty score to help identify false positives [32]. Nevertheless, because the reads resulting from index misassignment showed even distribution across the virus or viroid genomes to which they matched, these measurements did not assist in flagging false positives. Therefore, the correct interpretation of pathogen detections for diagnostics must include a check for the presence of index cross-talk among samples multiplexed and sequenced in the same experiment. We implemented a Contamination Flag (CF) and tested it on the direct read annotation approaches (Kodoja and PVDP) for the RNA-Seq data, and VirReport and VirusDetect for the sRNA-Seq data. The minimum detection threshold (0.1, 1.0, or 10%) depended on the dataset and thus required optimization for each tool and HTS approach. The CF enabled to detect all false positives in the RNA-Seq data with the exception of one call in the SP2 and two calls in the SP1 data. The abundance of viroids was much more variable in SP1 for viroids, preventing a clear separation of false positives from true positives. All index misassignments detected by VirReport and VirusDetect were flagged in the sRNA-Seq data.

While the CF is a helpful tool to make sense of the data generated, false positive predictions would still need to be independently confirmed by a secondary method (e.g., PCR). For this reason, we would strongly recommend using library prep chemistries (e.g., UDIs and UMIs) and technologies (e.g., non-patterned flow-cells) to mitigate the risks of index misassignment that jeopardize both the sensitivity and the potential rapid turnaround times of HTS-based diagnostics approaches. Finally, positive and negative controls should also be included at different stages of the HTS test to further guide in the accurate detection of contamination which may have occurred during sample preparation and sequencing. For example, an ‘alien’ control (i.e., a plant host harboring a microbial organism which is known not to be associated with the samples tested) can be included to check for cross-contamination between samples (Sébastien Massart, personal communication).

Looking at overall performance, for the RNA-Seq dataset, the direct read annotation approaches were more sensitive at detecting an HSVd present at low titer than the de novo assembly tools in our study. These latter failed to assemble sequences into contigs due to the presence of low abundant quasi-genomes in the host plant, as has been reported elsewhere [57]. Another advantage of direct read annotation over the de novo assembly methods is the ease of installation and rapid execution. However, their sensitivity also makes direct read annotation methods more prone to detect false positives (e.g., cross-talk, detection of endogenous viruses). Therefore, they can be used as first-pass screening tools to identify viral sequences and guide downstream bioinformatics procedures such as reference-guided assembly, as pointed out by Gutierrez et al. [32]. In comparison, de novo assembly-based approaches enable assembling complete viral genomes and thus provide a better resolution in complex cases such as multiple related viruses co-infecting the same plant, discriminating recombination events, detecting sequence divergence, or identifying new viral species.

A critical consideration for total RNA extraction is the presence of highly abundant host plant RNAs that can limit the detection sensitivity of viruses. Ribosomal RNA depletion is now a standard process to reduce the sequencing of non-informative RNAs. However, this approach is not yet readily available to produce sRNA-Seq data. Among our samples, we found an effective removal of rRNAs in RNA-Seq, accounting, on average, for 10% of sequenced reads with a maximum of 30%. In comparison, up to 60% of the reads in sRNA-Seq samples encoded rRNA fragments. We observed variability in the amount of rRNA-derived sRNAs across plant species; peach, raspberry, and strawberry were the plant commodities with the least rRNAs in the sRNA fraction. The ribosomal RNA content is highly variable across plants, with differences observed in their rRNA gene repertoire and copy number [58]. The presence of rRNAs and other non-informative RNAs such as chloroplast, mitochondria, and tRNAs can yield varying profiles of informative sequences even when the number of raw sequencing reads might seem adequate. Therefore, we think that assessing and measuring the amount of available informative reads for HTS-based diagnostics provides a better evaluation of the suitability of available data for pathogen diagnostics and decision making.

## 5. Conclusions

HTS methods, ribodepleted RNA-Seq and small RNA-Seq, detected all true positive plant viruses and viroids across various plant commodities diagnosed by PEQ assays. The differences between HTS methods were noticeable when reporting false-positive detections. Selection of library prep chemistry, sequencing technology, and bioinformatics methods can impact the reporting of sample cross-contaminations. In this study, small RNA-Seq combined with a de novo assembly approach using specifically virus-derived siRNAs shows the best overall performance detecting all known plant viral pathogens with minimal or no cross-talk events, making it a suitable approach for quarantine testing at PEQ.

## Figures and Tables

**Table 1:** Selected PEQ positive control plants infected with known viruses and viroids. Presence of regulated viruses detected using molecular (PCR and ELISA) and bioassays (biological and woody indexing).

Sample ID	Commodity	Species	Positive Detections in PEQ
MT001	Citrus	Citrus Troyer × Frost-Lisbon	CEVd
MT002	Prunus	*Prunus persica*	PNRSV
MT003	Citrus	*Citrus* × *aurantiifolia* (Christm.) Swingle	CTV, CVEV, CDVd, HSVd
MT004	Citrus	*Citrus medica* L.	CEVd, CTV, HSVd
MT005	Raspberry	*Rubus idaeus*	RBDV
MT007	Citrus	*Citrus* sp.	CDVd, HSVd
MT008	Citrus	*Citrus sinensis*	CDVd, HSVd
MT010	Ornamental grass	*Miscanthus sinensis* ‘Morning light’	Novel potyvirus (MsiMV)
MT011	Citrus	*Citrus medica* L.	CTV, CVd-VI, HSVd
MT012	Iris	*Iris* sp. ‘Crimson colossus’	ISMV, TRSV
MT013	Strawberry	*Fragaria vesca* ‘Alpine’	SMoV
MT014	Strawberry	*Fragaria vesca* ‘UC4’	SMoV
MT015	Strawberry.	*Fragaria* sp.	SMoV
MT016	Sweet potato	*Ipomoea batatas*	SPFMV

CEVd = Citrus exocortis viroid, CTV = Citrus tristeza virus, CVEV = Citrus vein enation virus, CDVd = Citrus dwarfing viroid, CVd-VI = Citrus viroid VI, HSVd = Hop stunt viroid, ISMV = Iris severe mosaic virus, PNRSV = Prunus necrotic ringspot virus, RBDV = Raspberry bushy dwarf virus, SMoV = Strawberry mottle virus, SPFMV = Sweet potato feathery mottle virus, TRSV = Tobacco ringspot virus. Species names shown in italics.

**Table 2:** Viral detection sensitivity for the different methods and technology tested in this study. Sensitivity was calculated as the number of true positives recovered divided by the total number of known targets identified by PEQ (ratio indicated in brackets). Targets included 24 viruses/viroids across 14 plant samples for the SP1 and 14 viruses/viroids across nine samples for the SP2.

Sequencing Provider	Sequencing Technology	Software	Sensitivity (%)
SP1	RNA-Seq	Kodoja	100 (24/24)
		PVDP	95.8 (23/24)
		SPAdes	95.8 (23/24)
		Trinity	95.8 (23/24)
	sRNA-Seq	VirusDetect	100 (24/24)
		VirReport-SPAdes	100 (24/24)
		VirReport-Velvet	100 (24/24)
SP2	RNA-Seq	Kodoja	100 (14/14)
		PVDP	100 (14/14)
		SPAdes	100 (14/14)
		Trinity	92.9 (13/14)
	sRNA-Seq	VirusDetect	100 (14/14)
		VirReport-SPAdes	78.6 (11/14)
		VirReport-Velvet	100 (14/14)

**Table 3:** Detection sensitivity and false discovery rate at specified subsampling depth for sRNA-Seq and RNA-Seq datasets. Sensitivity was calculated as the number of true positives (number of correctly identified viruses and viroids) divided by the total number of known target species identified by PEQ (ratio indicated in brackets). Targets included 13 viruses and 11 viroids across 14 plant samples for the SP1 and 9 viruses and 5 viroids across nine samples for the SP2. The discovery rate was calculated as the number of false positives (indicated in brackets) divided by the total number of both true positives and false positives detected.

Sequencing Technology	Subsampling	Viruses		Viroids	
		Sensitivity (%)	False Discovery Rate (%)	Sensitivity (%)	False Discovery Rate (%)
RNA-Seq SP1 (Kodoja)	1 M	100 (13/13)	66.7 (26)	72.7 (8/11)	0
	2.5 M	100 (13/13)	71.7 (33)	72.7 (8/11)	0
	4 M	100 (13/13)	73.5 (36)	81.8 (9/11)	0
	5 M	100 (13/13)	75.9 (41)	81.8 (9/11)	0
	10 M	100 (13/13)	78.3 (47)	100 (11/11)	8.3 (1)
	All reads	100 (13/13)	80.6 (54)	100 (11/11)	21.4 (3)
sRNA-Seq SP1 (VirReport-Velvet)	1 M	92.3 (12/13)	7.7 (1)	100 (11/11)	8.3 (1)
	2.5 M	100 (13/13)	7.1 (1)	100 (11/11)	15.3 (2)
	4 M	100 (13/13)	13.3 (2)	100 (11/11)	21.4 (3)
	All reads	100 (13/13)	48 (12)	100 (11/11)	52.1 (11)
RNA-Seq SP2 (Kodoja)	1 M	100 (9/9)	25 (3)	80 (4/5)	0
	2.5 M	100 (9/9)	35.7 (5)	100 (5/5)	0
	4 M	100 (9/9)	43.8 (7)	100 (5/5)	0
	5 M	100 (9/9)	50.0 (9)	100 (5/5)	0
	10 M	100 (9/9)	57.1 (12)	100 (5/5)	0
	All reads	100 (9/9)	69.9 (20)	100 (5/5)	0
sRNA-Seq SP2 (VirReport-Velvet)	1 M	100 (9/9)	0	80 (4/5)	0
	2.5 M	100 (9/9)	0	100 (5/5)	16.7 (1)
	4 M	100 (9/9)	0	100 (5/5)	16.7 (1)
	All reads	100 (9/9)	0	100 (5/5)	16.7 (1)

## Data Availability

The data presented in this study are openly available in the Short Read Archive (SRA) database under the BioProject PRJNA752836 (https://dataview.ncbi.nlm.nih.gov/sra/PRJNA752836, accessed on 19 December 2021).

## Supplementary Materials

The following supporting information can be downloaded at: https://www.mdpi.com/article/10.3390/biology11020263/s1. Supplementary Materials Figure S1. Overview of experimental design and data analysis using two HTS methods and two service providers. Supplementary Materials Figure S2. VirReport workflow overview. The VirReport pipeline has been implemented in nextflow for its easy access and reproducible and scalable use. The end-to-end VirReport pipeline uses quality filtered small RNA-Seq data as input. Read sequences are selected based on size (e.g., 21–22 nt or 24 nt-long reads) for de novo assembly and subsequent scaffolding. Scaffolds are then screened against nucleotide and protein databases. A summary report is generated using BlastTools.jar and custom scripts. Coverage and other statistics are provided to assist with the interpretation of results. Consensus genome sequences, which incorporate the variants of each detected viruses and viroids, can optionally be derived using bedtools (https://bedtools.readthedocs.io/en/latest/content/overview.html, accessed on 9 March 2021) and bcftools [59]. Importantly, VirReport flags potential cross-sample contamination events based on all multiplexed samples in the same HTS experiment. A plus sign (+) indicates tools and workflows users can add to the pipeline as by default these are not part of the automated pipeline. Supplementary Materials Table S1. Metadata of positive control plant samples. The table details the plant commodity and the species name (when available) from which the sample was derived; the tissue type from which RNA was extracted; the RNA quality assessments performed: RNA integrity number (RIN) score and 28 S/18 S rRNA (28 S/18 S) ratio; and which sequencing providers the samples were sent for sequencing. Supplementary Materials Table S2. Post-entry quarantine molecular assays performed on control plants used in this study. PCR primers and amplification conditions for each assay are summarized along with reference publications from which the assays were implemented. BSA = Bovine Serum Albumin, ssRNA(+) = positive single-stranded RNA. Supplementary Materials Table S3. General statistics of the sequencing runs derived with RNA-Seq and sRNA-Seq by SP1 and SP2. The table shows the starting number of reads that were initially sequenced, and the read count and read percentage obtained after removing low-quality reads. rRNA filter = number of remaining reads after removing sequences with sequence similarity to ribosomal RNAs. (A) SP1 RNA-Seq. (B) SP1 RNA-Seq. (C) SP1 sRNA-Seq. (D) SP2 sRNA-Seq. SD = standard deviation, MIN = minimum, MAX = maximum. Supplementary Materials Table S4. De novo assembly statistics. (A) Total number of assembled scaffolds using SPAdes and Trinity for ribodepleted RNA-Seq data. Similarly total number of assembled scaffolds using VirReport and VirusDetect tools using small RNA-Seq data are shown. All numbers are counts after merging initially assembled contigs with CAP3. (B) Total number of scaffolds with sequence similarity to viruses or viroids for both ribodepleted RNA-Seq or small RNA-Seq datasets approaches are shown. NT = not tested. Supplementary Materials Table S5. BLASTN results of assembled virus and viroid sequences obtained with RNA-Seq and sRNA-Seq. The searches were performed using megablast against the non-redundant NT database (SPAdes, Trinity and VirReport) and against a virus database plant_239_U100 (VirusDetect). The BLASTN results were summarized using BlastTools. The table shows the best reference hit recovered for each species. sacc = subject (reference) accession sequence, slen = subject (reference) length, coverage = percent of the reference genome covered by the assembled sequence(s), av-pident = average sequence identity percent of assembled sequence(s) to reference, ND = not detected. * VirusDetect outputs a virus table which provides coverage and % identity values. Supplementary Materials Table S6. Total reads mapping to consensus sequences derived from RNA-Seq and sRNA-Seq de novo assemblies. * We used the best homology hit (KT692938.1) for ISMV as we only obtained partial sequences matching this virus genome. ^ If more than one virus or viroid strain was recovered in a sample, mapping was only performed against the most abundant strain. FPKM = fragments per kilobase of transcript per million mapped reads, NT = not tested. Supplementary Materials Table S7: Virus table and implementation of a contamination flag (CF) for RNA-Seq results derived for SP1 using KODOJA. The threshold used for the CF was [(RMP/Max_RPM)*100] to be at least 1%. Read counts was derived from the stringent level assignment for viruses (i.e., same assignment designated by Kraken and Kaiju) and the non-stringent read assignment for viroids (as viroids do not have protein assignments in RefSeq). True positives are highlighted in blue, false positives are highlighted in grey. Two false positive viroid calls were missed by the CF and are indicated in red. RPM = reads per million; RPM_MAX = highest observed RPM for a given virus/viroid in the same multiplexed sequencing run. Supplementary Materials Table S8: Virus detections and implementation of a contamination flag (CF) for RNA-Seq results derived for SP2 using KODOJA. The threshold used for the CF was ≥1%. True positives are highlighted in blue, false positives are highlighted in grey. RPM = reads per million; RPM_MAX = maximum observed RPM for a given virus/viroid in the same multiplexed sequencing run; TP = true positive. Supplementary Materials Table S9: Virus detections and implementation of a contamination flag (CF) for RNA-Seq results derived for SP1 using PVDP. The threshold used for the CF was ≥ 0.1%. True positives are highlighted in blue, false positives are highlighted in grey. RPM = reads per million; RPM_MAX = highest observed RPM for a given virus/viroid in the same multiplexed sequencing run; TP = true positive. Supplementary Materials Table S10: Virus detections and implementation of a contamination flag (CF) for RNA-Seq results derived for SP2 using PVDP The threshold used for the CF was ≥0.1%. True positives are highlighted in blue, false positives are highlighted in grey. One false positive virual call was missed by the CF and is indicated in red. RPM = reads per million; RPM_MAX = highest observed RPM for a given virus/viroid in the same multiplexed sequencing run; TP = true positive. Supplementary Materials Table S11: Coverage statistics and implementation of a contamination flag (CF) for sRNA results derived for SP1 using VirReport-SPAdes on 21 nt- and 22 nt-long reads. The threshold used for the CF was ≥1%. True positives are highlighted in blue, false positives are highlighted in grey. Mean coverage = mean coverage in bases to the genome/sequence of the best homology match, PCT_1X = fraction of bases that attained at least 1X sequence coverage, PCT_10X = fraction of bases that attained at least 10X sequence coverage, PCT_20X = fraction of bases that attained at least 20X sequence coverage, RPM = reads per million, sacc = accession number of best homology match recovered. TP = true positive. Supplementary Materials Table S12: Coverage statistics and implementation of a contamination flag (CF) for sRNA results derived for SP1 using VirReport-Velvet on 21 nt- and 22 nt-long reads. The threshold used for the CF was ≥1%. True positives are highlighted in blue, false positives are highlighted in grey. Mean coverage = mean coverage in bases to the genome/sequence of the best homology match, PCT_1X = fraction of bases that attained at least 1X sequence coverage, PCT_10X = fraction of bases that attained at least 10X sequence coverage, PCT_20X = fraction of bases that attained at least 20X sequence coverage, RPM = reads per million, sacc = accession number of best homology match recovered. TP = true positive. This table does not include the CVD-VI call detected by VirReport-Velvet in sample M010 in the de novo assembly derived using 24 nt-long reads and reported in Figure 4 and Table 3. Supplementary Materials Table S13: Coverage statistics and implementation of a contamination flag (CF) for sRNA results derived for SP2 using VirReport-Velvet on 21 nt- and 22 nt- long reads. The threshold used for the CF was ≥1%. True positives are highlighted in blue, false positives are highlighted in grey. Mean coverage = mean coverage in bases to the genome/sequence of the best homology match, PCT_1X = fraction of bases that attained at least 1X sequence coverage, PCT_10X = fraction of bases that attained at least 10X sequence coverage, PCT_20X = fraction of bases that attained at least 20X sequence coverage, RPM = reads per million, sacc = accession number of best homology match recovered. TP = true positive. Supplementary Materials Table S14: Virus table and implementation of a contamination flag (CF) for sRNA results derived using VirusDetect for SP1 on 21 nt- and 22 nt-long reads. The threshold used for the CF was [(RMP/Max_RPM)*100] to be at least 1%. True positives are highlighted in blue, false positives are highlighted in grey. RPM = reads per million, TP = true positive. Supplementary Materials Table S15: Virus table and implementation of a contamination flag (CF) for sRNA results derived using VirusDetect for SP2 on 21 nt- and 22 nt-long reads. The threshold used for the CF was ≥1%. True positives are highlighted in blue, false positives are highlighted in grey. RPM = reads per million, TP = true positive.
