# Supplementary material for: Side-by-Side Comparison of Post-Entry Quarantine and High Throughput Sequencing Methods for Virus and Viroid Diagnosis
Source: Biology (Basel). 2022 Feb 8;11(2):263. doi: 10.3390/biology11020263 (PMC8868628; doi:10.3390/biology11020263)
Supplement: Supplementary file 1 [file biology-11-00263-s001.zip › biology-1539752-supplementary.pdf]

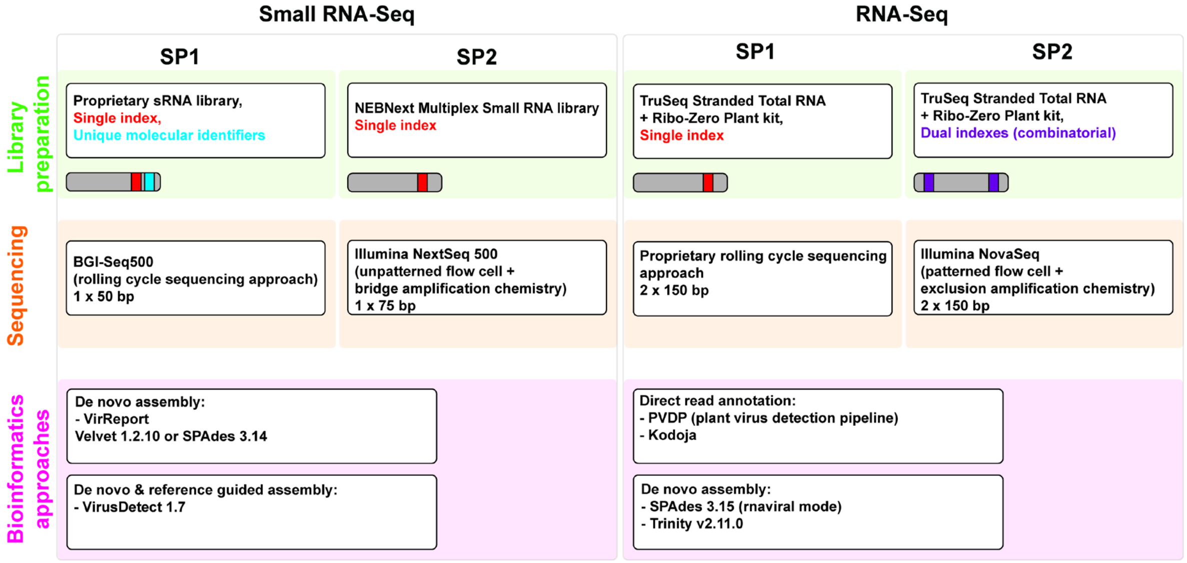

Figure S1. Overview of experimental design and data analysis using two HTS methods and two service providers.

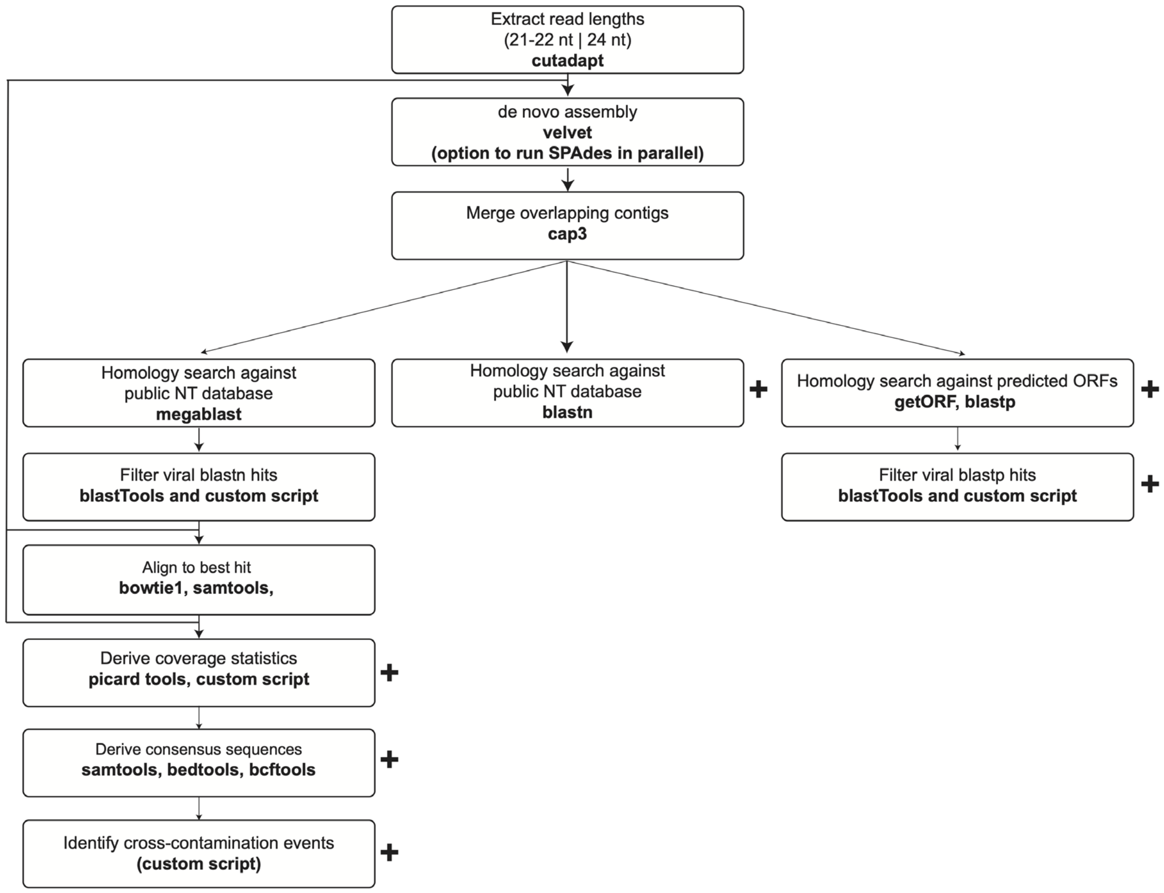

Figure S2. VirReport workflow overview. The VirReport pipeline has been implemented in nextflow for its easy access and reproducible and scalable use. The end-to-end VirReport pipeline uses quality filtered small RNA-Seq data as in-put. Read sequences are selected based on size (e.g., 21–22 nt or 24 nt-long reads) for de novo as-sembly and subsequent scaffolding. Scaffolds are then

screened against nucleotide and protein databases. A summary report is generated using BlastTools.jar and custom scripts. Coverage and other statistics are provided to assist with the interpretation of results. Consensus genome sequences, which incorporate the variants of each detected viruses and viroids, can optionally be derived using bedtools (https://bedtools.readthedocs.io/en/latest/content/overview.html ac-cessed on 9 March 2021) and bcftools [59]. Importantly, VirReport flags potential cross-sample contamination events based on all multiplexed samples in the same HTS experiment. A plus sign (+) indicates tools and workflows users can add to the pipeline as by default these are not part of the automated pipeline.
